# Cardiac muscle regulatory units are predicted to interact stronger than neighboring cross-bridges

**DOI:** 10.1038/s41598-020-62452-7

**Published:** 2020-03-26

**Authors:** Mari Kalda, Marko Vendelin

**Affiliations:** Laboratory of Systems Biology, Department of Cybernetics, School of Science, Tallinn University of Technology, 12618 Tallinn, Estonia

**Keywords:** Computational biophysics, Computational models, Cardiovascular biology

## Abstract

Strong interactions between cross-bridges (XB) and regulatory units (RU) lead to a steep response of cardiac muscle to an increase in intracellular calcium. We developed a model to quantitatively assess the influence of different types of interactions within the sarcomere on the properties of cardiac muscle. In the model, the ensembles consisting of cross-bridge groups connected by elastic tropomyosin are introduced, and their dynamics is described by a set of partial differential equations. Through large scans in the free energy landscape, we demonstrate the different influence of RU-RU, XB-XB, and XB-RU interactions on the cooperativity coefficient of calcium binding, developed maximal force, and calcium sensitivity. The model solution was fitted to reproduce experimental data on force development during isometric contraction, shortening in physiological contraction, and ATP consumption by acto-myosin. On the basis of the fits, we quantified the free energy change introduced through RU-RU and XB-XB interactions and showed that RU-RU interaction leads to ~ 5 times larger change in the free energy profile of the reaction than XB-XB interaction. Due to the deterministic description of muscle contraction and its thermodynamic consistency, we envision that the developed model can be used to study heart muscle biophysics on tissue and organ levels.

## Introduction

Cooperative interaction of cross-bridges and regulatory proteins during contraction of the heart is a manifestation of the principle "The whole is greater than the sum of its parts”. Due to interactions between cross-bridges (XB) and regulatory units (RU) in cardiac muscle, the heart can relax at resting state calcium levels and develop tension when the intracellular calcium concentration increases during a twitch^1^. The mechanisms behind cooperative contraction are debated with several possible alternatives considered, ranging from neighbor interactions in different forms (RU-RU, XB-XB, XB-RU)^2^ to interactions over the sarcomere^3,4^. To test these hypotheses quantitatively, mathematical modeling approaches have been developed ranging from simple set of ODEs^2^ to large-scale models using Monte Carlo techniques simulating up to half a sarcomere^5–7^. Through analyses of the simulated data, the contribution of different types of interactions have been predicted to have different effects on formation of Ca^2+^ sensitivity and the maximal force development by the muscle^2,7^. However, most of the analyses have been done on steady-state kinetics^2,4,8,9^ and sometimes extended to isometric contractions^5,7^. There is a lack of non-empirical models that take into account physiological shortening contractions and, in addition to the developed force, energy consumption of the muscle. Our earlier works^10–12^ and this study are addressing this gap and assessing the contribution of cooperative interaction mechanisms, when dynamics of physiological contractions and energy consumption by the muscle are taken into account.

A tight link between energy consumption of the heart and mechanical work is well characterized through pressure-volume loops. It has been shown that oxygen consumption in the heart can be related to pressure-volume area (PVA) or, in the case of a tissue, stress-strain-area (SSA)^13,14^. The PVA is a specific area in the pressure-volume (PV) diagram surrounded by the end-systolic pressure volume line, the end-diastolic line and the systolic segment of the PV trajectory for heart contraction. The models based on Huxley formalism have the ability to link developed mechanical force during contraction with biochemistry in thermodynamically consistent way by taking into account the relationship between cross-bridge force and its free energy^15^. While providing a framework to describe muscle contraction, taking into account microscopic reversibility, the description of cooperativity using Huxley-type models have been problematic^16^. Recently, we developed a new approach that allowed us to describe cooperativity using Huxley-type models^12^. In short, instead of studying a single cross-bridge, we formulated equations describing the dynamics of a group of cross-bridges. We assumed that all cross-bridges in the group are connected by tropomyosin and, by considering tropomyosin as an elastic string, we could estimate the influence of neighboring cross-bridges on the free energy of a cross-bridge group. This description allowed us to reproduce experimental data for isometric contraction and the linear relationship between ATP consumption and SSA at different contraction modes. While we were able to demonstrate that cooperativity can be studied by this approach, the main limitation in the developed description was that the simulation results demonstrated a relatively low cooperativity of calcium binding in steady-state, significantly smaller than the recorded values^17,18^.

The aim of this work was to reproduce the cooperativity of Ca^2+^ activation of actomyosin interactions and estimate the contribution of these interactions to the cooperative action of the muscle. For that, we developed a mathematical model of cardiac muscle on the basis of the theoretical framework considering ensembles of cross-bridges^12^, fitted the measurements from different types of experiments, and assessed the resulting free energy profile of the reactions.

## Theory

An overview of Huxley-type cross-bridge models and detailed description of including cooperativity into the model is given in^12^. Here, we describe only changes that were introduced in this work, allowing us to reproduce the high cooperativity of calcium binding in cardiac muscle.

To overcome low cooperativity of Ca^2+^ binding by our original model^12^, we targeted one specific assumption used in the simulations of the model. Namely, we considered cross-bridge ensembles as a group of five consecutive cross-bridges, with the first and the last cross-bridge in the group always in the unbound state. In this work, we allow the first and the last cross-bridge to bind calcium and interact with actin with the only limitation of an imposed periodic boundary condition. As we show below, this change in the boundary conditions leads to a major simplification of the model and allows to describe cooperative contraction of ensembles composed of *n* cross-bridges by performing simulations on only one representative cross-bridge.

**Figure 1:**
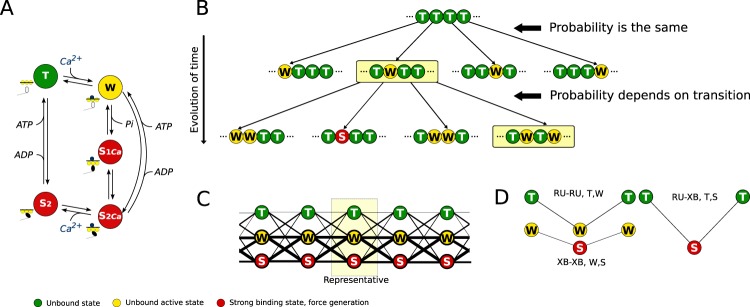
Scheme of mathematical model with cooperativity. (**A**) Acto-myosin interaction is represented by five-state cross-bridge model. In this description, we have two biochemical states where no force is generated (T and W) and three force-generating states (S_1Ca_, S_2Ca_, and S_2_). Out of these states, W, S_1Ca_, and S_2Ca_ have Ca^2+^ bound to associated troponin-C. (**B**) Example transformations of cross-bridge ensemble that demonstrate differences in transition probabilities induced by deformation of the string connecting cross-bridges. While binding of Ca^2+^ to cross-bridges in the top row configuration is the same, the transitions of each cross-bridge in the second row would depend on the state of the cross-bridge neighbors. (**C**) As all cross-bridges have the same distribution between the states in the infinite string, the state of the string is reflected by the representative cross-bridge. In this example, the cross-bridges were assumed to be distributed as 15% in state T, 45% in W, and 40% in S. The interactions between neighbor cross-bridges are shown by lines between them with the thickness of the line proportional to the probability to observe this interaction. (**D**) Three types of cooperative interactions considered in this work with the corresponding index of the free energy change shown next to the corresponding deformation. See the main text for details.

In this work, as in the earlier one^12^, each cross-bridge is assumed to be in one of the five states (Fig. 1A). This representation associates cross-bridge and troponin-C Ca^2+^ binding sites, which allows us to follow transitions imposed by calcium binding as well as cycling of cross-bridges between strong and unbound binding states in a relatively simple manner. To introduce cooperativity, we assume that cross-bridges form groups, with the interaction between cross-bridges influenced by an elastic string connecting them, representing tropomyosin^12^. The binding of Ca^2+^ or formation of strong cross-bridges will shift tropomyosin^19,20^ and, through that, influence neighboring cross-bridges.

Let us illustrate how the periodic boundary condition and ability of the boundary cross-bridges to participate in contraction will change the mathematical formulation of the cooperativity of cross-bridge ensembles. As an initial condition, we consider all cross-bridges detached and without Ca^2+^ bound to troponin C (Fig. 1B, top). Assuming that the cross-bridges are aligned on an infinitely long string, Ca^2+^ binding to any of them (transition from state T to W) will have the same probability for any of the cross-bridges on the string. This is shown as transitions from the top state to the different states in the second row of Fig. 1B. After that transition, the further evolution of the cross-bridge will depend on the state of its immediate neighbors, as shown in the second transition on Fig. 1B. Let us split this infinite string of cross-bridges into groups of 5 and assume that the cross-bridges together with RUs on the boundaries can also perform transitions between states using the same kinetics as the cross-bridges in the middle of the group. When compared to the cross-bridges in the middle of the group, the only difference for a boundary cross-bridge is that one of its immediate neighbors is from another side of the group. Through such connection between cross-bridges on both sides of the group, periodicity is imposed.

The combination of the initial condition imposing the same state on all cross-bridges and the periodic boundary condition for the ensemble of 5 (or any other number) cross-bridges leads to a major simplification – we can consider that all cross-bridges in the ensemble have the same distribution among its states (T, W, and S states). Indeed, there is nothing to discriminate between the cross-bridges in top of Fig. 1B, as they all have the same initial conditions and follow the same transitions, when considering the state of their immediate neighbors. As a result, the probability of finding a particular cross-bridge in a certain state is independent of the cross-bridge location in the infinite string. Thus, if we know the distribution of states for a cross-bridge, we also know the corresponding distributions for the neighboring cross-bridges and, as a result, we can find the distribution of the states of the cross-bridge with its immediate neighbors by multiplying the corresponding probabilities. For example, motif W-S-T will have probability *p*_*W*_ ⋅ *p*_*S*_ ⋅ *p*_*T*_ if all three cross-bridge motifs are considered with *p*_*i*_ being a probability of a cross-bridge to be in state *i*.

The resulting representation of cooperative interaction between cross-bridges is depicted as an example in Fig. 1C. In this example, the group of 5 cross-bridges is shown with the most of the cross-bridges in W or S state and some of the cross-bridges are next to RU’s without bound Ca^2+^ (state T). As the distribution of cross-bridge states is the same for the whole string, the probability of having neighboring connections can be calculated and is shown in the figure by the thickness of the lines connecting the cross-bridges (Fig. 1C). In this configuration, it is clear that connections W-W, S-S, W-S are the main observed ones. However, there is still a probability to observe T-W, T-T, and T-S (thin lines in Fig. 1C).

To quantify the extent of cooperative interaction, we specified an increase in elastic free energy for a string connecting RUs and cross-bridges. When taking free energy of a string with the neighbors in the same configuration (W-W, T-T, and S-S) equal to zero, the cooperativity was described by a free energy increase when the neighboring states were T-W (RU-RU interaction, Fig. 1D, top left), W-S (XB-XB interaction, Fig. 1D, bottom left), and T-S (RU-XB interaction, Fig. 1D, right). The corresponding free energy changes were *U*_T;W_, *U*_W;S_, and *U*_T;S_, respectively. On Fig. 1D, all interactions were shown as symmetric, with the left and the right neighbor in the same state and the central cross-bridge in some other state. In this case, the corresponding free energy change would be double, as the change is summed up for the left and the right connection (2 ⋅ *U*_T;W_ for example in Fig. 1D, top left). As the neighboring interactions can be of different types, they sum up to give the full change in the free energy induced by cooperativity. In Fig. 1D, we illustrate the relative deformation of the elastic string in the different cooperative interaction modes as suggested on the basis of our simulations (described in Results). Note that the predicted angles are smaller, but are shown in correct proportions relative to each other.

According to the assumption that tropomyosin connects all cross-bridges in a group, the elastic deformation of tropomyosin will influence the free energy of the group as well as the reaction kinetics. In the considered five-state cross-bridge model, there are three different positions where tropomyosin can shift: T, W, S. For simplicity, as shown in^12^, we can describe the free energy changes in the group induced by tropomyosin deformation through two parameters: *U*_T;W_, *U*_W;S_. The free energy change induced by other combinations can be described using these free energy changes after making some assumptions regarding the geometry of the system^12^.

To sum up the theoretical part, we made changes in the boundary conditions and the treatment of the boundary cross-bridges when compared to the example model in^12^. As a result of these changes, in this work, we are simulating infinitely long strings consisting of cross-bridges/RUs that interact via closest neighbor interaction. From a modeling point of view, the model tracks only one cross-bridge and assumes that the neighboring cross-bridges have the same distribution between their states. This leads to the governing equation for the state *A*

1 $$\frac{\partial {n}_{A}}{\partial t}+\frac{\partial {n}_{A}}{\partial x}v(t)=\mathop{\sum }\limits_{i}^{{\mathbb{S}}}\mathop{\sum }\limits_{B}^{{\mathbb{S}}}\mathop{\sum }\limits_{j}^{{\mathbb{S}}}({k}_{i,B,j\_i,A,j}{n}_{i}{n}_{B}{n}_{j}{C}_{B,A}(t)-{k}_{i,A,j\_i,B,j}{n}_{i}{n}_{A}{n}_{j}{C}_{A,B}(t))$$

where *k* is the rate constant for the transition of the cross-bridge from state *A* to *B*, which depends on the states of the neighboring cross-bridges; *v* = *v*(*t*) is the rate of the half-sarcomere lengthening; and the influence of calcium is represented through factor *C*_*A*,*B*_(*t*), as in^12^. Here, the state of the cross-bridges is described by fractions *n*_*A*_(*x*, *t*) of cross-bridges that are in the same state *A* at time *t* in the subensemble at *x*^12,15^. Transitions between all possible states of a single cross-bridge (${\mathbb{S}}$) are formally considered with the rate constants set to zero if the corresponding transition does not occur according to the kinetic scheme (Fig. 1A).

## Results

### Ca^2+^ binding at isometric steady-state conditions

**Figure 2:**
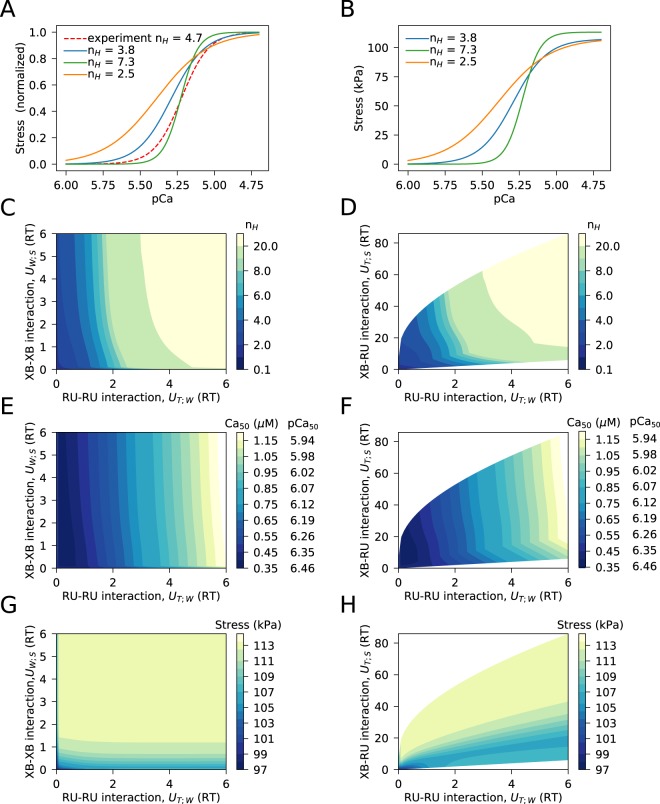
Cooperativity of muscle contraction studied on the basis of calcium binding relationship. All simulations were performed at the half-sarcomere length of 1.1 *μm*. (**A,B**) Steady state stress development at different Ca^2+^ concentration (normalized in **A** and absolute values in **B**). The difference at cooperativity coefficient is achieved by changing the values of free energy of tropomyosin deformation. Here, the simulation results are compared with the Hill relationship fit of experimental data from Gao *et al*.^18^. The used free energies describing cooperativity were as follows: *n*_*H*_ = 3.8 was obtained with *U*_*T*;*W*_ = 0.0*RT*, *U*_*W*;*S*_ = 2.0*RT*; *n*_*H*_ = 7.3 with *U*_*T*;*W*_ = 2.0*RT*, *U*_*W*;*S*_ = 1.8*RT*, and *n*_*H*_ = 2.5 with *U*_*T*;*W*_ = 0.4*RT*, *U*_*W*;*S*_ = 0.4*RT*. (**C**) Simulation results for estimating the range of possible cooperativity coefficient *n*_*H*_ values, by varying free energies of tropomyosin deformation *U*_T;W_ and *U*_W;S_ corresponding to RU-RU and XB-XB interactions, respectively. Note that the model simulations cover the range of physiological values for *n*_*H*_. (**D**) Since XB-RU interaction presented by free energy change *U*_T;S_ is geometrically linked in the model with *U*_T;W_ and *U*_W;S_, the same data, as in (**B**), is shown as a dependency of *n*_*H*_ on *U*_T;W_ and *U*_T;S_. (**E–H**) Ca^2+^ sensitivity (shown by Ca_50_ using concentration and pCa on **E**,**F**) and maximal developed stress (**G,H**) as a function of free energy changes induced by tropomyosin deformation.

By introducing periodic boundary conditions for cross-bridge ensembles, we are able to reproduce the cooperativity of muscle contraction. The model solutions are able to reach cooperativity levels that cover a physiologically relevant range (Fig. 2A,B). In these simulations, we varied two principal free energies that define the extent of tropomyosin deformation: *U*_T;W_, *U*_W;S_. These free energy differences are the changes in free energy induced by the deformation of the elastic string segment between two neighboring cross-bridges when the cross-bridges are in states T-W (*U*_T;W_) or W-S (*U*_W;S_), compared to the energy of the segment when the cross-bridges are in the same state. Thus, by increasing *U*_T;W_ and *U*_W;S_, we can increase the cooperativity of the cross-bridges leading to an increase in the cooperativity coefficient, as demonstrated in Fig. 2C. Since we relate *U*_T;S_ to *U*_T;W_ and *U*_W;S_ through geometrical considerations of tropomyosin deformation^12^, the same relationship as in Fig. 2C can be plotted as a relationship between *U*_T;W_ and *U*_T;S_ (Fig. 2D).

The free energy changes induced by tropomyosin deformation are a manifestation of the different types of cooperative interactions between cross-bridges (XB) and troponin-tropomyosin regulatory units (RU). Namely, *U*_T;W_ would correspond to RU-RU interaction, *U*_W;S_ and *U*_T;S_ to XB-XB and XB-RU, respectively. Thus, according to the simulation results in Fig. 2C,D, RU-RU and XB-RU interactions have the largest effect on the cooperativity coefficient *n*_*H*_.

The cooperative interactions between cross-bridges and regulatory units in the muscle have different effects on Ca^2+^ sensitivity and developed stress. While Ca^2+^ sensitivity is mainly regulated through RU-RU interaction (*U*_T;W_ in Fig. 2E,F), maximal developed force is sensitive to XB-XB interactions (*U*_W;S_ in Fig. 2G,H). Note how the half-saturation Ca^2+^ concentration level (Ca_50_) is mainly sensitive to *U*_T;W_ change (Fig. 2E) and how *U*_W;S_ is the main axis along which the maximal developed stress is changing (Fig. 2G).

### Simulations of twitch dynamics

**Figure 3:**
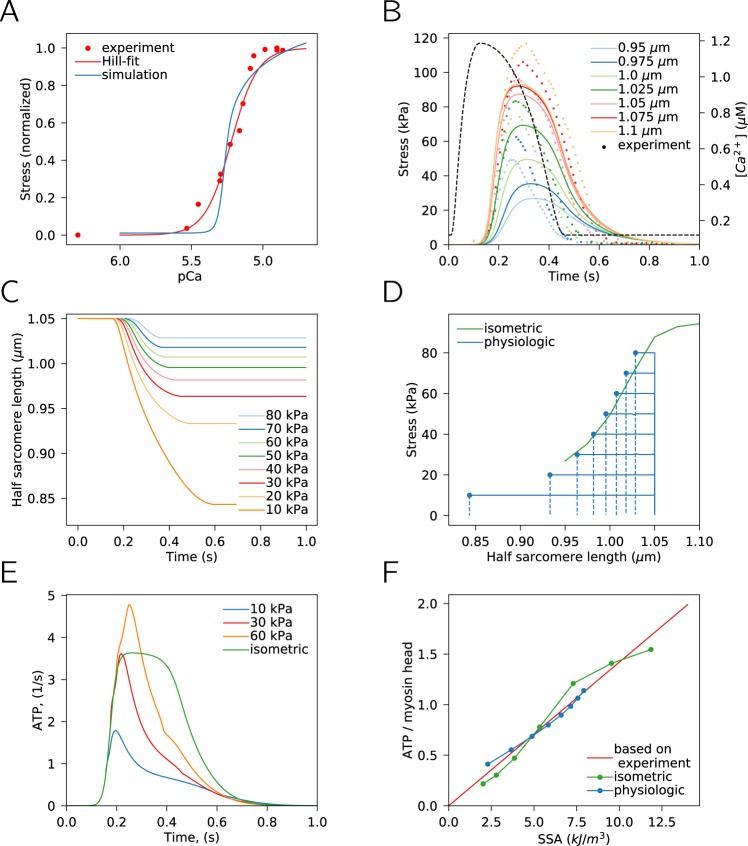
Simulation results obtained after fitting model solution against experimental data using a single set of model parameters. (**A**) Calculated (blue) normalized steady-state stress at the different Ca^2+^ concentrations at half-sarcomere length 1.1 *μm* compared with the experimental results from^18^. The experimental data is shown as measured values (dots) and the Hill equation fit (red line). (**B**) Isometric contraction as a function of time at different half-sarcomere lengths (see inset) and Ca^2+^ concentration transient used in simulations (dashed black line). (**C**) Change in sarcomere length during physiological contraction at different afterloads (see inset). (**D**) End-systolic line for isometric contraction (maximal developed stress-strain relationship) compared to changes in stress and strain during physiological contractions at different afterloads. Systolic (solid lines) and diastolic (dashed lines) phase of physiological contractions are shown. Note that end-systolic points for physiological contraction (points) are close to the end-systolic line for isometric contraction. (**E**) ATP consumption by a cross-bridge during isometric and physiological contractions at different workloads at half-sarcomere length 1.05 *μm*. Note how ATP consumption depends on afterload. (**F**) Total amount of consumed ATP molecules per myosin head during a cardiac cycle as a function of stress-strain area (see text for explanation) for isometric and physiological contractions compared with the 65% efficiency relationship which is based on experimental data. Note that both contraction modes reproduce the linear relation.

After demonstrating the model’s ability to reproduce the cooperativity of Ca^2+^ binding at isometric steady-state conditions, we proceeded with the analysis of model performance during a twitch. For that, we fitted the model solution against experimental data on force development, sarcomere deformation and ATP consumption by cross-bridges in different loading scenarios (see residuals section in Methods). The fitted results achieved using the developed five-state model with the same set of model parameter values are shown at Fig. 3. As described below, while the obtained model solutions are not perfectly matching the experimental data, they capture several aspects of cardiac function.

In Fig. 3A, the calculated steady state stress development at different Ca^2+^ levels is shown against the measurements^18^. The value of the cooperativity coefficient for the simulation results was 6.6 (Fig. 3A), which is larger than the value estimated from the experimental data (4.7, red line in Fig. 3A). While the value of the cooperativity coefficient was in the high end of the reported values, it ensured that the muscle was able to relax in the diastolic phase at a non-zero Ca^2+^ concentration in the cell.

As a model prediction, i.e. without any fitting of this particular parameter, we calculated the fraction of myosin heads bound to actin (duty ratio) at steady-state, as in Fig. 3A. In our simulations, at 1 *μ*M Ca^2+^, the duty ratio was ~ 7.5%, in agreement with the experimental recordings performed on isolated myosin heads^21^.

The simulation results showing stress development and sarcomere dynamics during isometric contraction are shown in Fig. 3B. In these simulations, as well as in the simulations using physiological contraction (described below), the Ca^2+^ concentration was assumed to follow the same transient (Fig. 3B, the dashed line). As shown in Fig. 3B, isometric stress in our simulation is developed after a significant delay, when compared with the Ca^2+^ transient. The reasons for the delay in the raise of force are not clear and are discussed below with the other study limitations. However, the model is able to reproduce the increase in isometric force development and the twitch prolongation with the increase of the sarcomere length, as required to reproduce the increase in ATP consumption with the increase of the sarcomere length (see below and discussion in^10^).

In addition to isometric contractions, we looked into the performance of the model in a "physiological” contraction protocol. In this protocol, at the beginning, the muscle develops force at constant sarcomere length. As soon as a given afterload is reached, contraction follows the isotonic mode, i.e. the muscle is allowed to shorten to keep the developed force equal to the afterload. The contraction is switched back to the isometric mode as soon as the muscle has to lengthen to keep the force equal to the afterload. The corresponding sarcomere change dynamics are shown in Fig. 3C for different afterloads. It is known that the end-systolic relationship between developed stress and strain is similar for isometric and shortening contractions^14^. This property has been fitted and reproduced by the model very well (Fig. 3D). In Fig. 3D, we show the maximal developed stress for isometric contractions and compare that to changes in stress related to strain during physiological contractions. As shown, the end-systolic points for physiological contractions are close to the strain-maximal stress relationship during isometric contractions, in accordance with the experimental data^14^.

For the model solutions describing isometric and physiologic contractions (Fig. 3A–D), we looked into the energy requirements of a single beat for isometric and physiological contraction protocols. For that, we followed the average detachment rate of the cross-bridges and calculated the rate of ATP consumption (Fig. 3E). As shown in Fig. 3E, the ATP consumption depends on the contraction protocol and its specific parameters, afterload in this case. In accordance with the experimental data^14^, the amount of ATP hydrolized per twitch is close to the expected linear relationship between ATP consumption and SSA (Fig. 3F). Note that the amount of ATP consumed by the myosin heads is the same for isometric and physiological contractions as long as the SSA for the corresponding contraction is the same (Fig. 3F), in agreement with^13^. In the simulations, we fitted the model results with the linear relationship between SSA and ATP consumption calculated for 65% contractile efficiency (red line in Fig. 3F). The contractile efficiency^13^ for the isometric contractions was 71% at maximal SSA taking into account the myosin ATPase concentration of 0.18 mM^22^ and free energy change during ATP hydrolysis of 60 kJ mol^−1^^13,23^. Our efficiency is similar to values estimated in experimental data, where the chemomechanical efficiency of cross-bridge cycling is in range of 60–70%^13^.

The minimized residual consisted of several parts, each contributing to the total residual value and reflecting some aspect of cardiac function. For the optimal set of model parameters, the largest contribution corresponded to the error in the fit of the ATP consumption and stress-strain area relationship (47% of total residual value, Fig. 3F). This was followed by the residual component describing the difference between end-systolic points for isometric and physiologic contractions (31%, Fig. 3D) and goodness of the fit of isometric contraction dynamics (22%, Fig. 3B). The contribution of the error in the fit of steady-state stress development at different Ca^2+^ concentration was rather small (<2%, Fig. 3A). Note, that the sum of the contributions was larger than 100% due to rounding errors.

### Free energy profile inducing cooperativity

**Figure 4:**
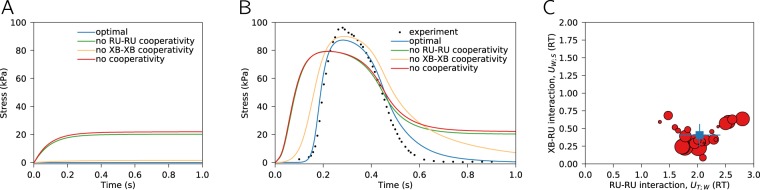
Analysis of tropomyosin-induced cooperativity and its influence on overall free energy profile of cross-bridge reactions. Effect of removing one of the cooperative interactions on calculated isometric stress (shown for half-sarcomere length 1.05 *μ*m) for simulations with steady-state Ca^2+^ of 0.12 *μ*M (**A**) and Ca^2+^ transient (**B**). Notice how removal of RU-RU, XB-XB, or all forms of cooperativity (RU-RU, RU-XB, XB-XB) changes the optimal solution with the rest of the model parameters staying the same. (**C**) Free energies defining extent of RU-RU (*U*_T;W_) and XB-XB (*U*_W;S_) induced cooperativity as found by fitting model solution against experimental data. Here, 25 solutions with the smallest residuals were selected and the corresponding free energies plotted as red dots. Size of the dot reflects the goodness of the fit with the largest dots corresponding to the best fits. Average free energy changes are shown by a square with the corresponding standard deviation. Note the larger influence of RU-RU interaction when compared to XB-RU interaction on cooperativity predicted by the model.

On the basis of the model fits, we could analyze the obtained model parameters with the focus on the free energy changes induced by tropomyosin deformation. According to our results, the cooperativity of the muscle contraction is mainly induced by RU-RU interactions, as indicated by *U*_T;W_ being significantly larger than *U*_W;S_. For the best fit, the corresponding free energy changes were ~ 2 RT (*U*_T;W_) and 0.2 RT (*U*_W;S_). On Fig. 4A,B, we demonstrate effect of removing cooperativity, either fully or in parts, on the calculated isometric stress. The simulations were performed using the same model parameters as in Fig. 3. The cooperativity affects the steady-state developed force (Fig. 4A) and its dynamics during a twitch (Fig. 4B). In both series of simulations, the cross-bridges were all in state T at time zero. As the Ca^2+^ concentration is non-zero at rest, in the absence of cooperativity or with low cooperativity, as in the calculations without RU-RU interactions, the model predicts a significant tension development at rest (stabilization of the solution in Fig. 4A). When looking into the dynamics of force development (Fig. 4B), in addition to the changes induced by the differences in steady-state of the solution (Fig. 4A), a lower cooperativity results in slower force increase and decrease. With optimal parameter values, as it is shown in Fig. 4A,B, removal of XB-XB interaction had significantly smaller effect on developed force than removal of cooperativity induced by RU-RU interaction.

To illustrate the variability in the optimal model parameters, we plotted the free energy changes corresponding to the best 25 fits in Fig. 4C (fit with the smallest residual is shown in Fig. 3). When the best 25 fits were analyzed, the values were 2.0±0.4 RT (*U*_T;W_) and 0.41±0.15 RT (*U*_W;S_). If we compare them formally by taking them as 25 measurements, then *U*_T;W_ is significantly larger than *U*_W;S_ (*p* < 0.001 in paired t-test, extremely strong evidence *BF* > 100 in Bayesian paired t-test). These free energies can be translated into the angles for the corresponding deformed state of the elastic string^12^. While this model is not as accurate as the models based on detailed molecular structures, the ratio of obtained angles can be compared with the experimental data. Our simulations predict that the deformation angle induced by binding of Ca^2+^ (corresponding to *U*_T;W_) is 1.5–1.7 ×  larger than the angle induced by binding of myosin (based on *U*_W;S_). This is similar to the structural studies predicting the corresponding ratio for azimuthal tropomyosin movements equal to ~ 2.5 ×  when based on electron microscopy data^24,25^, or ~ 2 × , when based on X-ray data^25^.

## Discussion

In this paper, we introduce a mathematical model of cardiac muscle that reproduces the cooperative interaction between cross-bridges and regulatory units during contraction. The model is based on a novel framework^12^ that treats groups of cross-bridges as ensembles and extends T.L. Hill’s^15^ approach to cooperative action of cross-bridges. The framework and the corresponding model is built up with strict thermodynamic consistency between simulated reactions and developed mechanical force, ensuring that the laws of thermodynamics are obeyed at each part of the model. The model reproduced cardiac muscle properties measured in stationary and dynamic conditions including isometric and shortening contractions. The model reproduced physiological integral properties such as contractile efficiency and the cooperativity coefficient of contractility. To our knowledge, it is the first time a cardiac muscle model is able to reproduce these data sets while being thermodynamically consistent. Our sensitivity analysis demonstrated a major impact of RU-RU interaction on Ca^2+^ sensitivity and, together with XB-RU interactions, on cooperativity during contraction. XB-XB interactions, on the other hand, were of major influence in modulation of developed stress. By fitting our model solution against experimental data, we quantified the free energy change introduced through RU-RU and XB-XB interactions, and showed that RU-RU interactions lead to ~ 5 times larger change in the free energy profile of the reaction than XB-XB interactions. Since the developed framework and the model lead to a set of deterministic equations, we envision that it can be used for studies of mechanical contraction on cellular, tissue, and organ levels.

### Periodic boundary conditions as a critical change in the model

When we developed the formalism for introduction of cross-bridge cooperativity into the Huxley-type models in a thermodynamically consistent manner, in the example model, we used cross-bridge ensembles consisting of five consecutive cross-bridges^12^. These ensembles were connected by an elastic string and, to simplify the model code development, we assumed that the first and the last cross-bridge were always without bound Ca^2+^ and were not producing force. Thus, we allowed only three out of five cross-bridges to attach and participate in the actomyosin interaction. As a result, these three cross-bridges were always "dragged” by the boundary cross-bridges into a detached state with unbound calcium. We hypothesized that the boundary conditions resulted in a low cooperativity coefficient found in our simulations and tested this hypothesis by assuming that the ensemble has a periodic boundary condition: the last cross-bridge in the ensemble can be described by the same distribution functions as the first one. As our simulations demonstrate (Fig. 2), this change allowed us to cover physiologically relevant cooperativity coefficient values and use the model to assess the contribution of RU-RU, XB-RU, and XB-XB in cooperative muscle contraction. In addition, by reproducing a high level of cooperativity, we were able to use measured Ca^2+^ concentration traces with significant Ca^2+^ levels during diastole and still get low developed stress, an observation highlighted by others before^1,7^.

The use of periodic boundary conditions led to a major simplification of the governing equations. In our simulations, the use of periodic boundary conditions allowed us to simulate only one cross-bridge in the ensemble with all other cross-bridges following the same probability distribution functions. In terms of equations, this simplification resulted in major reduction of the number of partial differential equations in the model. As we use a 5-state description for cross-bridge kinetics (Fig. 1A), after the simplification, we can simulate only one representative cross-bridge leaving us with 5 partial differential equations that have to be solved. Such simplification of the governing equations reduces significantly the computing requirements and allows to use the model for detailed analysis of free energy profiles of reactions.

### Limitations of the study

As every mathematical model, our model is based on multiple assumptions. The assumptions are discussed in details in the study where we introduced the formalism of handling muscle cooperativity in a thermodynamically consistent manner using Huxley-type models^12^. These assumptions include single binding site for each cross-bridge, reduction of partial differential equations dimensions by assuming that the position of neighbor cross-bridges is the same, and considering only small ensembles of cross-bridges^12^. The latter limitation has been lifted in this work and replaced with periodic boundary conditions imposed on the cross-bridge ensembles. As is common in the Huxley-type models, we ignored the elasticity of actin filaments^26^ and possible heterogeneity in thin filament strain^27^. We are planning to work on the limitations of the model while keeping it as simple as possible.

When we fitted the model parameters against experimental data or overall properties of the cardiac muscle, we were not able to reproduce all measurements with the same set of model parameters. In particular, although the model is able to reproduce the cooperativity coefficient in a physiological range, the simulation results for stress development during isometric contractions show a delay between the Ca^2+^ transient and stress development. In addition, time to peak and relaxation time of the isometric stress development calculated by the model were larger than the measured data suggests. Also, the tension-pCa curve does not fit the experimental data that well.

We can only speculate at this point on how to improve the model fits. Possibly, the overall minimization of the residual has to be implemented differently to obtain better fits. At present, a large fraction of the overall residual is contributed by the stress-strain area and the ATP consumption relationship (Fig. 3F). Taking into account that the model can reproduce the relationship reasonably well, maybe better overall fits will be obtained if the weight factor used for that part of the residual is changed. We may have to develop new functions to describe rate constants of transitions between the cross-bridge states. When comparing to the study by Aboelkassem *et al*.^28^, our kinetic scheme lacks the strong cross-bridge formation ability in the absence of Ca^2+^. While we consider Ca^2+^ detachment in the strong state (transition from S_2Ca_ to S_2_ in Fig. 1A) and the following transition to the unbound binding state T, the transition is linked to ATP hydrolysis and is biased towards the cross-bridge detachment. In this respect, adding Ca^2+^-independent thin filament activation by allowing formation of state S_1_ from T, tuning the free energy change associated with transitions between Ca^2+^-free states when compared to Ca^2+^-binding states as well as associated kinetic constants, may improve the fit. Since the main focus for this work was to demonstrate that we can reproduce the cooperativity coefficient at a level consistent with experimental data, we have left the work on improving the goodness of the fit to further studies.

### Relative contribution of RU-RU, XB-RU, XB-XB interactions

Before discussing the contributions of different types of cooperativity, a few important aspects of the analysis have to be highlighted. By associating cooperativity with the deformation of tropomyosin, we could clearly separate free energy changes induced by each reaction into a "cooperative” and an "individual” part. Namely, the cooperative interaction induced changes in free energies through deformation of the tropomyosin (free energy changes *U*_T;W_, *U*_W;S_, and *U*_T;S_) while changes in free energy induced by change of the state were attributed to the individual part. For example, during Ca^2+^ binding, the free energy difference *G*_*W*_ − *G*_*T*_ was associated with the individual part while the corresponding change in free energy of tropomyosin, dependent on the neighbor cross-bridges, was associated with the cooperative part. Thus, through the free energy changes *U*_T;W_, *U*_W;S_, and *U*_T;S_ we could characterize the cooperative action in muscle contraction. When compared to the recent work on cooperativity by Land and Niederer^7^, these free energy changes would correspond to changes in tropomyosin stiffness and/or tropomyosin displacement angle in their model.

The next aspect is related to the use of a mechanical model behind the analysis of cooperative interactions. Tropomyosin was modeled as an elastic string with a given stiffness and pre-defined deformation states allowing us to incorporate the energy of mechanical deformation into the free energies of the ensemble states. This is similar to several studies on modeling of cooperativity^5,29,30^. As derived earlier^12^, our geometrical model resulted in a relationship between *U*_T;W_, *U*_W;S_, and *U*_T;S_ with one of the free energies determined by the other two. The use of a defined mechanical model behind cooperative interaction allowed us to ensure that the thermodynamic consistency of the model is always fulfilled. However, as a result of the link between free energy changes, when we analyzed the effects of RU-RU and XB-XB interactions, we also changed the free energy of the corresponding XB-RU interaction. On the other hand, we are sure that we study cooperative interaction effects and not possible effects induced by violation of the laws of thermodynamics.

The analysis presented on Fig. 2 clearly shows the different effects of different types of cooperative interaction. RU-RU and XB-RU determine the level of cooperativity between Ca^2+^ concentration changes and the developed force. The effects of RU-RU and XB-RU interaction on the cooperativity has been noted earlier^2^, with the others predicting minor role of XB-RU on the cooperativity^7^. In contrast to the earlier works, we varied the free energy changes in a systematic manner over a large range of combinations. For example, in^7^, while binding constants were varied to see their influence on cooperativity, tropomyosin stiffness influence was not studied in as much detail as the corresponding free-energy change analysis in our work.

In agreement with Razumova *et al*.^2^, we demonstrated that RU-RU cooperative interactions can have a major influence on muscle Ca^2+^-sensitivity without changing the Ca^2+^ binding constants (Fig. 2E). However, we did not observe a major effect of RU-RU interactions on the maximal developed force. The force was mainly influenced by XB-XB over a large number of tested combinations (Fig. 2G). This has been suggested by other studies as well^2,7^.

As for XB-RU interaction, we have not observed its effect on Ca^2+^ sensitivity (mainly RU-RU according to our results) or maximal developed force, as suggested in^7^. In the case of Ca^2+^ sensitivity, the changes were all almost orthogonal to the XB-RU-corresponding axis on Fig. 2F. The maximal force did change on Fig. 2H, but that was in accordance with the changes in XB-XB cooperativity, since the variability in force was along *U*_*W*;*S*_ isolines in *U*_T;W_ − *U*_W;S_, and *U*_T;W_ − *U*_T;S_ planes (Fig. 2G,H). When comparing with others^7^, the importance of XB-RU interaction on Ca^2+^ sensitivity was suggested on the basis of the simulations, where the myosin binding constant was varied. Variation of the myosin binding constant would correspond to changes of free energy difference between states W and S_1Ca_ (Fig. 1A), which is the individual part of the free energy change and is not directly associated with the changes induced by cooperative interactions between cross-bridges and regulatory units through tropomyosin deformation. In addition, changes in the myosin binding constant have to be consistent with the available free energy of ATP hydrolysis. On basis of the model description in^7^, the absence of ATP hydrolysis-induced changes in cross-bridge free energy suggests that the corresponding thermodynamic constraint was not taken into account in the simulations, where the myosin binding constant was varied. Thus, the difference in the influence of XB-RU interactions can be caused by differences in compared free energy changes (cooperative interaction vs binding constants) and/or a possible violation of thermodynamic constraints during variation of myosin binding constants in^7^.

After analysing the sensitivity of force development on cooperative interactions between cross-bridges and regulatory units, we estimated the free energy changes that would be consistent with a set of steady-state and dynamic experiments covering different aspects of cardiac force generation and energy consumption. We found that free energy changes induced by RU-RU interaction are expected to be 5 ×  larger than XB-XB interaction (Fig. 4). As explained in Results, the free energy changes are in the agreement with the tropomyosin deformation predicted by electron microscopy and X-ray studies^24,25^.

### Thermodynamic constraints linking mechanics and energetics

The use of T. L. Hill formalism^15^ ensured that the mechanical work performed by the cross-bridges is in agreement with the invested energy through ATP hydrolysis. In addition to our earlier model on cooperativity^12^, the same thermodynamically consistent approach was used by Tanner *et al*. in their Monte Carlo method based models^31,32^. As a result of the link between mechanical force and chemistry, our model does not have thermodynamics-imposed limitations on shortening and isometric contractions. This is in sharp contrast with the models, where the mechanical force is found by multiplying the population of cross-bridges in a force-producing state with a factor^5,7,9,29^. While such multiplication does not violate thermodynamic constraints in simulations of isometric contraction (no work is done when the length of the muscle is unchanged at the level considered by the model), this assumption can be invalid as soon as any work is performed. Indeed, when the work produced is not constrained by ATP hydrolysis energy, one can easily imagine *perpetuum mobile*-type scenarios, where a cross-bridge would be linked to ATP synthase and drive it mechanically to produce more ATP than is used by the cross-bridge. Thus, the predictions of such simplified models and their use to study contraction dynamics have to be carefully assessed to ensure that the used parameters and obtained model solutions are within the thermodynamic constraints.

### Organ-level simulations

We envision that the model and the corresponding framework presented in this work can be used to study the effects of cooperativity not just on single cell or tissue levels, but on the organ level as well. The developed model is deterministic and can be used as a drop-in replacement of ODE models used in these types of studies. For reference, simulation times with the current model using a single thread were from ~ 30 seconds (isometric) to 8 minutes (physiologic contraction with 40 kPa afterload) on the computational node. While ODE-based models^33,34^ are usually considered for organ-level modeling, despite the possible inconsistencies with thermodynamic constraints as discussed above, PDE-based models can be successfully used to study mechanics and energetics of the heart as well. Already 19 years ago, we performed a study using the Huxley-type model^10^ to describe muscle properties in the underlying model of the heart muscle wall deformation^11^. In our experience, the main numerical problems were imposed by finding the deformation rate field and were not related to the use of the Huxley-type models. In particular, during simulations, the solutions of the Huxley-type model were easily run in parallel on a cluster at each iteration making that part of simulations non-rate limiting.

*In conclusion*, the presented framework and the model reproduce the cooperativity of cardiac muscle contraction and predicts that the changes in free energy induced by RU-RU interaction are about 5 times larger than XB-XB interaction.

## Methods

Detailed description of used formalism and mathematical methods is given in^12^. Here, we describe only the changes made in this work.

### Model description

In our simulations, we consider a five-state cross-bridge model, where T and W are detached states, and S_1Ca_, S_2Ca_ and S_2_ are attached states (Fig. 1A). The model considers cross-bridges forming groups and describes the dynamics of the group ensembles. By using the periodical boundary conditions, we can describe our system by five PDE equations, as explained in the Theory part.

#### Calcium dynamics

Calcium dynamics are introduced into the model through a phenomenological description that mimics measured calcium transients. For that, we introduced factor *C*_*A*,*B*_(*t*) that depends only on states A and B and can be written as

2 $${C}_{{\rm{A}},{\rm{B}}}(t)=\left\{\begin{array}{ll}Ca(t) & \,{\rm{if}}\,({\rm{A}},\,{\rm{B}})\in \{\left({\rm{T,W}}\right),\left({{\rm{S}}}_{2},{{\rm{S}}}_{2{\rm{Ca}}}\right)\},\\ 1 & \,{\rm{if}}\,\ ({\rm{A}},\,{\rm{B}})\in \left\{\left({\rm{W}},{{\rm{S}}}_{1{\rm{Ca}}}\right)\right.,\\  & \left.\left({{\rm{S}}}_{1{\rm{Ca}}},{{\rm{S}}}_{2{\rm{Ca}}}\right),\left({\rm{W}},{{\rm{S}}}_{2{\rm{Ca}}}\right),\left({\rm{T}},{{\rm{S}}}_{2}\right)\right\},\\ 0 & \,{\rm{otherwise}}\,.\end{array}\right.$$

Here, *Ca*(*t*) describes Ca^2+^ transient as follows

3 $$Ca(t)=C{a}_{0}+C{a}_{p}\ \ast \ \left\{\begin{array}{ll}{e}^{\left(1-\frac{1}{1-\eta {(t)}^{2}}\right)} & \,{\rm{if}}\,| \eta (t)|  < 1\\ 0 & \,{\rm{otherwise,}}\,\end{array}\right.$$

where *Ca*_0_ is diastolic calcium, *Ca*_*p*_ is amplitude and function *η*(*t*) is defined as follows:

4 $$\eta (t)=\left\{\begin{array}{ll}-1 & \,{\rm{if}}\,t < {t}_{0}\\ \frac{t-{t}_{p}}{{t}_{p}-{t}_{0}} & \,{\rm{if}}\,{t}_{0}\le t\le {t}_{p}\\ \frac{t-{t}_{p}}{{t}_{e}-{t}_{p}} & \,{\rm{if}}\,{t}_{p}\le t\le {t}_{e}\\ 1 & \,{\rm{if}}\,t < {t}_{e}\end{array}\right.$$

where *t*_0_ is start time for calcium transient, *t*_*p*_ is time to peak, and *t*_*e*_ is end of calcium transient.

Note, that in contrast to earlier works, where calcium was modeled via function changing from zero to one during a beat, we here use a function that has a significant non-zero value during the resting state of cardiomyocytes.

### Fitting and numerical methods

#### Residual

The model parameters were found by fitting the model solution to experimental data^18,35^ and properties of cardiac muscle. To estimate the fit, we used the least squares method. The corresponding residuals were divided into four parts

5 $$R={R}_{I}+{R}_{II}+{R}_{III}+{R}_{IV}.$$

where *R*_*I*_, *R*_*II*_, *R*_*III*_ are, respectively, the residuals for isometric force transients during a beat, for isometric and physiological end-systolic points comparison, and for achieving a linear relationship between *SSA* and energy consumption in isometric and physiological contraction. Calculation of the residuals is explained in^12^.

The fourth part of residuals was obtained by comparing the experimental^18^ and calculated Ca^2+^ sensitivity of the developed stress in steady-state. To cover the Ca^2+^ concentrations uniformly, we fitted the experimental data^18^ with the Hill equation and used the Hill relationship as an approximation of experimental points, that were fitted by the model solution. The stress computed by the model was normalized by its value at a Ca^2+^ concentration of 1.27 mM. Thus, the fourth part of residuals was:

6 $${R}_{IV}=\mathop{\sum }\limits_{i=1}^{{N}_{Ca}}{\left(\frac{{F}_{exp}(C{a}_{i})-{F}_{ca}(C{a}_{i})}{\gamma \sqrt{{N}_{Ca}}}\right)}^{2},$$

were *F*_*exp*_ is the Hill relationship fit to the measured stress from^18^, and *F*_*ca*_ is the normalized steady state stress computed by the model for the corresponding calcium concentration *Ca*_*i*_. The number *N*_*Ca*_ of used residual points was 100 with the residual normalization constant *γ* taken equal to 0.1.

**Figure 5:**
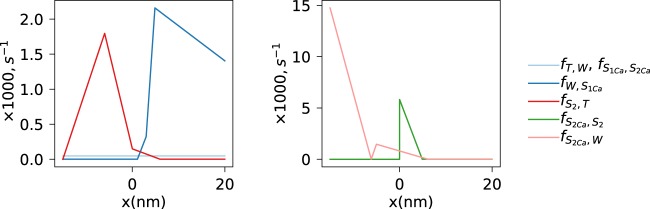
Piece-wise linear functions describing rate constants of the cross-bridge transformation reactions.

The parameter values set during simulations or found by fitting are shown in Table 1 and Fig. 5.

**Table 1:** Model parameters. Parameters marked with a "*” were optimized during fitting. In addition to the parameters shown in this table, the rate constants on Fig. 5 were fitted.

Parameters	Value	Optimized	Description
**Contractile element**			
K	0.56 *RTnm*^−2^		the Hooke constant^40^
d	36 nm		the distance between neighboring actin binding sites for a cross-bridge^41^
m	0.18 mol m^−3^		the myosin ATPase concentration,^22^
${{\rm{K}}}_{{\rm{tr}}}$	21.6 pN/nm		stiffness of a single tropomyosin^42^
${{\rm{l}}}_{\min }$	1.6 *μm*		the minimum sarcomere length^43^
${{\rm{l}}}_{\max }$	2.2 *μm*		the maximum sarcomere length
**Calcium dynamics**			
Ca_0_	0.12 *μM*		diastolic calcium concentration
Ca_p_	2.9 *μM*		calcium oscillation amplitude
t_0_	0.002 s	*	start time for calcium transient
t_p_	0.123 s	*	time to peak for calcium transient
t_e_	0.485 s	*	end of calcium transient
**Free energy parameters**			
G_T_	0		Free energy at state T, reference state
G_Ca_	1.06 RT	*	Free energy change induced by Ca release
G_W_	G_T_ − G_Ca_		Free energy at state W
G_S1Ca_	− 2 RT	*	Minimum free energy at state S_1Ca_
G_S2Ca_	− 21 RT	*	Minimum free energy at state S_2Ca_
G_S2_	G_S2Ca_ + G_Ca_		Minimum free energy at state S_2_
G_ATP_	24.04 RT		Free energy of ATP hydrolysis
x_1_	3 nm	*	Position of S_1_ minimum
x_2_	0 nm		Position of S_2_ minimum
U_WS_	0.23 RT	*	Free energy of tropomyosin deformation
U_TW_	2.01 RT	*	Free energy of tropomyosin deformation

#### Optimization procedure

As the model is sensitive to the changes in free energy profile, we were not able to use straightforward least squares minimization with common algorithms. Namely, when using common algorithms, such as the Levenberg-Marquardt algorithm^36^, some free energies were pushed too far during the minimization, leading to trivial solutions (as having no developed stress) and an inability to recover from it in further steps. To avoid such issues with minimization, we split the model parameters into two sets (named *scan* and *opt* sets) and optimized these sets as follows. For *scan* model parameters, we specified possible values for each of the parameters. This gave us a set of model parameters values consisting of all possible combinations for model parameters in *scan* set. For each of these combinations, we found an optimal set of model parameters in *opt* that would minimize the total residual. To find these optimal values, we used the Levenberg-Marquardt algorithm^36^, as after the partitioning of model parameters into two sets, it was able to minimize the residual.

The optimal model parameters were found by comparing the residuals obtained for each combination of the model parameter values in *scan*. The smallest obtained residual corresponded to the optimal model parameters set, consisting of a combination of model parameters from *scan* and *opt* sets.

The *scan* set consisted of three parameters describing free energy profiles: the value ${G}_{{{\rm{S}}}_{1}}^{min}$ and displacement *x*_1_ of minimal free energy in state S_1Ca_; the minimal free energy ${G}_{{{\rm{S}}}_{2}}^{min}$ in state S_2_. Note that the displacement *x* corresponding to the location of the S_2_ minimum was taken as an origin.

The *opt* set consisted of the rest of the optimized parameters. It included the parameters describing piece-wise linear functions of rate constants of the cross-bridge transformation reactions shown in Fig. 5. Taking into account microscopic reversibility of reactions, we have six independent cross-bridge cycling rates for every cross-bridge displacement. We assumed that Ca^2+^ association and dissociation rate constants were the same regardless of whether the cross-bridge was in a strong or unbound binding state (transitions between W and T or S_2Ca_ and S_2_) leading to only one independent rate constant required to describe the binding. In addition, this set included free energy changes induced by tropomyosin deformation (*U*_W;S_ and *U*_T;W_), Ca^2+^ transient time constants (*t*_0_, *t*_*p*_ and *t*_*e*_), and the free energy of Ca^2+^ binding.

#### Cooperativity coefficient

Cooperativity coefficient was found by fitting the data with the Hill equation:

7 $$F=({F}_{max}{[{{\rm{Ca}}}^{2+}]}^{{n}_{H}})/({[C{a}_{50}]}^{{n}_{H}}+{[{{\rm{Ca}}}^{2+}]}^{{n}_{H}}),$$

were *F*_*max*_ is maximal stress, *Ca*_50_ is [Ca^2+^] at half *F*_*max*_, and *n*_*H*_ is the Hill coefficient.

#### Numerical methods

The partial differential equations were discretized by a first order finite-difference method applied for displacement coordinate with the steps of 1 nm. The resulting system of ordinary differential equations was solved using the LSODE package^37^. To speed up simulations, the original LSODE routines were modified to take into account the sparsity of the system and the properties of the Jacobian matrix.

For isometric contractions, the simulations were performed as an integration of a system of ordinary differential equations. The solution was recorded for each 5 ms and used in the calculation of residuals.

For physiological contractions, the simulation were performed in three stages. In the first stage, the isometric contraction was calculated until reaching a given afterload. On approaching the afterload, a solution was requested every 1 ms, down from an interval of 5 ms for numerical stability of the solution. When the isometric solution led to a developed stress larger than the requested afterload, the simulations were continued in the isotonic stage.

In the isotonic stage, the sarcomere lengthening rate *v*(*t*) was found by assuming that the rate is constant for each integration time interval. The rate was varied by *hybrd* solver from MINPACK package^38^, until the calculated stress at the end of the time interval was the same as the given afterload, with a tolerance of 10^−5^. The procedure was repeated for the next time interval by taking the solution found at the end of the previous time interval as an initial condition.

The time interval was taken initially to 1 ms and was reduced 4 times in the case of failure of the nonlinear solver to find the sarcomere lengthening rate that would satisfy the imposed condition (calculated stress at the end of integration step equal to the given afterload). The reduction was continued until the integration interval was smaller than 5 ⋅ 10^−10^ ms. If that happened, the solution was considered unsuccessful. In the case of reduced time interval, the interval was increased if five successive iterations were successful. The maximal allowed time interval was set to 5 ms throughout the simulations and was not increased above it.

The isotonic stage continued until the sarcomere lengthening rate was found to be positive, i.e. corresponding to sarcomere lengthening. In this case, the simulations were switched to the isometric mode and were performed with *v*(*t*) set to zero. For this transition to occur, the interval was set at maximum of 1 ms. If the sarcomere lengthening stage was approached with interval larger than that, the interval was reduced before the transition.

For unsuccessful simulations, a large penalty was given in residual calculations, equal to approximately 200.

The model parameters were found by minimizing the least squares residuals. As described above, the optimization was performed using the Levenberg-Marquardt algorithm^36^. Simulations were performed on a cluster of Linux/Intel Xeon E5-2630L computers.

### Statistics

Statistical tests were performed using JASP. Here, we used paired samples t-test and Bayesian paired samples t-test. For Bayes Factor (BF) interpretation, common evidence categories were used^39^.
